# Does Suspected Sleep Disordered Breathing Impact on the Sleep and Performance of Firefighting Volunteers during a Simulated Fire Ground Campaign?

**DOI:** 10.3390/ijerph13020173

**Published:** 2016-01-29

**Authors:** Sarah M. Jay, Bradley P. Smith, Samantha Windler, Jillian Dorrian, Sally A. Ferguson

**Affiliations:** 1 Central Queensland University, Appleton Institute, Wayville 5034, South Australia; b.p.smith@cqu.edu.au (B.P.S.); samantha.windler1980@gmail.com (S.W.); sally.ferguson@cqu.edu.au (S.A.F.); 2 Centre for Sleep Research, University of South Australia, Adelaide 5000, South Australia; jill.dorrian@unisa.edu.au

**Keywords:** firefighters, sleep, performance, fatigue, sleep disordered breathing, obstructive sleep apnea

## Abstract

Adequate sleep is fundamental to workplace performance. For volunteer firefighters who work in safety critical roles, poor performance at work can be life threatening. Extended shifts and sleeping conditions negatively impact sleep during multi-day fire suppression campaigns. Having sleep disordered breathing (SDB) could contribute further to sleep deficits. Our aim was to investigate whether those with suspected SDB slept and performed more poorly during a fire ground simulation involving sleep restriction. Participants, *n* = 20 participated in a 3-day-4-night fire ground simulation. Based on oximetry desaturation index data collected during their participation, participants were retrospectively allocated to either a SDB (*n* = 8) or a non-SDB group (*n* = 12). The simulation began with an 8 h Baseline sleep (BL) followed by two nights of restricted (4 h) sleep and an 8 h recovery sleep (R). All sleeps were recorded using a standard electroencephalography (EEG) montage as well as oxygen saturation. During the day, participants completed neurobehavioral (response time, lapses and subjective fatigue) tasks. Mixed effects ANOVA were used to compare differences in sleep and wake variables. Analyses revealed a main effect of group for Total sleep (TST), REM , wake after sleep onset (WASO) and Arousals/h with the SDB group obtaining less TST and REM and greater WASO and Arousals/h. The group × night interaction was significant for N3 with the SDB group obtaining 42 min less during BL. There was a significant main effect of day for RRT, lapses and subjective fatigue and a significant day × group interaction for RRT. Overall, the SDB group slept less, experienced more disturbed sleep and had poorer response time performance, which was exacerbated by the second night of sleep restriction. This could present a safety concern, particularly during longer campaigns and is worthy of further investigation. In addition, we would recommend promotion of awareness of SDB, its symptoms and potential impact among volunteers and relevant agencies.

## 1. Introduction

Landmark studies in the field of sleep loss clearly demonstrate that restricted sleep, especially if occurring over consecutive nights is associated with marked impairment in daytime performance [1,2]. From a workplace perspective therefore, adequate sleep is fundamental to performance and is especially important for those who have a safety critical role where poor performance on the job can be life threatening. Volunteer firefighting is a prime example where poor performance can impact the safety of both the workers and their community but they are an under-researched occupational group in terms of sleep and performance at work. Due to the unpredictable and intense physical and environmental nature of wildfire suppression, volunteer firefighters work extended shifts (12–15 h) that often exclude driving times to and from the fire ground [3,4,5]. Such long work hours restrict opportunity for rest between shifts and indeed sleep restriction (<6 h) has also been reported [3,6].

Sleeping conditions during large-scale wildfire suppression may also negatively impact sleep. In Australia, volunteer firefighters can be deployed to remote areas across the country and be required to sleep on-site for days at a time. In these circumstances, volunteers typically sleep in communal tents as part of temporary camps [3]. While efforts are made to minimize the impact of exogenous factors on sleep, conditions are less than ideal with heat, light and noise all identified as factors impacting on sleep in on-site, tent accommodation [3].

In addition to workplace factors, there may be non-work related factors influencing sleep in these volunteers. Health status, such as having sleep disordered breathing (SDB) will impact sleep regardless of work or sleeping conditions but the impact of SDB has not been investigated in volunteer firefighters before. When untreated, SDB such as Obstructive Sleep Apnea (OSA—the most common breathing related sleep disorder) can result in sleep that is more fragmented with less slow wave sleep (SWS or N3) and less Rapid Eye Movement (REM) sleep [7]. The impact of OSA can extend into many aspects of life including, daytime functioning, mood and quality of life [8,9,10].

Unpublished data from a multi-day fire ground simulation conducted by our research group, conservatively estimated that nearly half of all participants (total *n* = 94) exhibited signs of SDB. The prevalence of OSA (as the primary sleep disorder) in Australia is, 4.7% [11] and while diagnosis was beyond the scope of the investigation, the proportion exhibiting SDB in some way is nonetheless concerning.

Of particular interest in the fire-fighting context is the combined impact of the work-related sleep restriction and the sleep disturbance resulting from untreated and/or undiagnosed SDB. This has not been specifically investigated in fire-fighters but there is general research suggesting that even when successfully treated, individuals with OSA are still more vulnerable to the performance effects of sleep restriction than healthy controls [12]. Our aim was to investigate sleep and performance of participants with suspected SDB during a 3-day-4-night fireground simulation that included two nights of sleep restriction. We predicted that those classified as demonstrating symptoms of SDB would obtain less total sleep, have a poorer quality of sleep (more arousals, awakenings), sleep architecture would be impacted (less REM and SWS/N3) and that they would rate their sleep as being of poorer quality across the 4-night protocol. Volunteers demonstrating symptoms of SDB would also have higher daytime fatigue scores and show poorer performance.

## 2. Experimental Section

Active volunteers of the South Australian Country Fire Service, Country Fire Authority (Victoria), Tasmania Fire Service, NSW National Parks and Wildlife Service, and ACT Fire and Rescue were invited to participate in a simulation of a 3-day, 4-night wildland fireground campaign. The aim of the simulation was to investigate the impact of key fireground conditions (heat and sleep restriction) on physical and neurobehavioural performance and sleep. The simulation included 4 study conditions but only the sleep restriction condition is presented here.

All participants gave their informed consent for inclusion before they participated in the study. Participants did not receive financial compensation for their participation in the study. The study was conducted in accordance with the Declaration of Helsinki, and the Human Research Ethics Committees at Central Queensland University (H12/01-016) and Deakin University (210-170).

### 2.1. Participants

Participants (*n* = 25) were allocated to the sleep restriction condition of the simulation. Based on analysis of Polysomnographic (PSG) and oximetry data recorded during their participation, participants were retrospectively allocated to either a Sleep disordered breathing (SDB) group or a non-SDB group (a detailed explanation of the criteria used is given below). Due to technical difficulties 5 participants did not have oximetry data and so were excluded from analyses. Thus the final analysis consisted of 20 participants (17 males, 3 females; aged 37 ± 13.7 years; Body Mass Index (BMI) of 29.1 ± 5.3 kg/m^2^). For SDB (n = 8), mean age and BMI were 44.9 ± 11.3 years and 32.6 ± 5.0 kg/m^2^ and for non-SDB (*n* = 12) 31.8 ± 13.0 years and 26.8 ± 4.2 kg/m^2^. Pre-Study Epworth Sleepiness Scale (ESS) [13] scores were 4.1 ± 2.8 and 3.8 ± 2.1 for SDB and non-SDB respectively.

### 2.2. Classification of Suspected Sleep Disordered Breathing

None of the participants had previously been diagnosed with a sleep disorder (according to self-report). Given that the study was not designed with clinical aims, nasal pressure and respiratory bands were not used. In the absence of these clinical measures the Oxygen Desaturation Index (ODI) was calculated, where ODI represented the number of times per hour of sleep that arterial oxygen saturation (measured via finger pulse oximetry) dropped by >3% from baseline [14,15,16,17,18]. Research has shown that in the absence of nasal pressure and respiratory effort, overnight oximetry is a valid diagnostic tool for OSA, especially when used in conjunction with PSG [14,15,16,17,18] and that an ODI of 10 would be a reasonable cut-off in the detection of at least moderate OSA [15,18].

Those participants who had two or more sleep periods (*i.e.*, half or more) with an ODI > 10 were allocated to the SDB group. In clinical settings, sleep and respiratory data from just one night is typically used in the diagnosis of SDB. Given the limitations in ODI measurement (desaturations only), a conservative approach was taken where two nights or more with an ODI > 10 was used as criteria for inclusion in the SDB group. [14,15,16,17,18]. The assumption from this point will be that the relevant group of participants is assumed to have SDB with an ODI pertaining to that of at least mild-moderate OSA.

### 2.3. Measures

Data was collected over six studies conducted at two different sites (Adelaide *n* = 4; Canberra *n* = 2). A portable sleep laboratory using the Siesta Portable EEG system (Compumedics, Melbourne, Victoria, Australia) was set up at each site. PSG data was recorded using the following recordings; two electroencephalography (EEG) channels (C3/M2 and C4/M1); two electro-oculographic (EOG) channels (left and right outer canthi), submental electromyogram (EMG), and arterial oxygen saturation (finger pulse oximetry). For the sleep records, 30 s epochs were manually assigned a sleep stage and arousals scored according to standard criteria [19]. The sleep scorer was blind to participant group. In addition, participants rated sleep quality on a 5-point scale (scale 1 = Very Well—5 = Very Poorly) following each sleep period.

The sleep variables investigated were; total sleep time (TST), sleep efficiency (TST/TIB * 100), Stage N1 minutes (N1), Stage N2 minutes (N2), Stage N3 minutes (N3), REM minutes (REM), Wake after sleep onset minutes (WASO), Arousals per hour, Sleep onset latency (SOL) and REM onset latency (ROL), Sleep Quality.

Subjective Fatigue using the Samn-Perelli Fatigue Checklist [20] and Response time using a 5-min palm-held psychomotor vigilance test (PVT) [21] were assessed at regular intervals throughout each day as part of a neurobehavioural test battery. As per standard practice, the reciprocal transformation of response time was used (RRT) to account for proportionality between the mean and standard deviation [22]. Lapses (response times >500 ms) are also reported. Prior to testing, participants completed three practice trials on the PVT to extinguish any learning effects.

### 2.4. Procedure

Participants spent four nights and three days in the simulation facility. All sleeps were recorded and during the daytime participants completed physical and neurobehavioural tasks aimed at replicating the type of performance required during wildfire suppression.

Participants arrived in the early evening and after a briefing, went to bed (time in bed (TIB) 8 h, 2230–0630) for the Baseline Sleep. This was followed by the Baseline Day where, following a Baseline neurobehavioural testing session at 10:30 A.M.. Full testing (including physical and physiological assessments) began at 12:30 and continued approximately 2-h till 18:30. Testing was scheduled at 12:30, 14:30, 16:30 with neurobehavioural testing occurring approximately 90 min into each session. TIB for Experimental Sleeps 1 and 2 (E1 and E2) was 4 h, (0200–0600). Testing began at 0800 on Experimental Days and concluded at 1830 (08:00, 10:00, 12:30, 14:30, 16:30). The final night was the Recovery Sleep (R), where TIB was again 8 h (2200–0600) (see [23] for greater detail and data from the daytime protocol).

For sleep periods all participants (between 4 and 5 per study group) slept in the same room, on stretcher beds with sleeping bags and pillows. This set up is representative of what is used during campaign wildfire suppression. Ambient temperature was maintained at 18–20 °C. A sleep technician monitored sleep signals from an adjacent room. Participants were given pagers so they could alert the technician for assistance during the night.

### 2.5. Statistical Analyses

All data were analysed using SPSS Version 21 (IBM, 2012) using mixed effects Analysis of Variance (ANOVA). As compared to traditional ANOVA, mixed effects models can deal with missing data and can better account for the correlation of multiple data points from the same participant over time [24].

For the Study (ODI and TIB) and Sleep variables, mixed effects ANOVA were constructed with *night* (BL, E1, E2, R) and *group* (SDB or non-SDB) entered as Fixed Effects and participant ID as a Random Effect to account for inter and intra individual differences. Models compared the main effect of *night*, *group* and *group* × *night* interaction.

On the Baseline Day, the study proper did not begin until 1230 and as a result, there was one less testing session on the Baseline day compared to the Experimental Days. For comparative purposes, four (of a possible 5) approximately time matched tests have been used in the analyses. For the wake variables (subjective fatigue, RRT and Lapses), mixed effects ANOVA were constructed with *day* (BL, E1, E2), *time* (0930^(E1, E2)^/1030^(BL)^, 1400, 1600, 1800) and *group* (SDB or non-SDB) entered as Fixed Effects and participant ID as a Random Effect to account for inter and intra individual differences. Models compared the main effects of *day*, *group*, *time* and the *group* × *day* and *day* × *time* 2-way interactions. With small samples, it becomes difficult to test for interactions. Accordingly, the 3-way interaction term (*group* × *day* × *time*) and one two-way interaction of little interest (*group* × test) were removed from the models.

## 3. Results

Results of mixed effects ANOVA for each of the study and sleep variables are summarised in Table 1.

**Table 1:** Summary of mixed effects ANOVA results for sleep and study variables.

Variables		Group			Night			Group × Night		
		df	F	p	df	F	p	df	F	p
Study	TIB	1, 15.8	0.22	0.645	3, 47.2	6601.12	<0.001	3, 47.2	3.48	0.023
	ODI	1, 17.2	16.38	0.001	3, 44.6	0.83	0.486	3, 44.6	1.09	0.364
Sleep	TST	1, 18.5	4.33	0.052	3, 47.9	158.73	<0.001	3, 47.9	1.65	0.190
	Efficiency	1, 18.1	4.18	0.056	3, 47.2	15.13	<0.001	3, 47.2	0.83	0.483
	Arousals/h	1, 18.4	6.58	0.019	3, 47.1	5.31	0.013	3, 47.1	0.18	0.914
	SOL	1, 64.0	0.73	0.398	3, 64.0	13.70	<0.001	3, 64.0	0.82	0.489
	ROL	1, 16.1	0.01	0.947	3, 46.6	14.99	<0.001	3, 46.6	0.78	0.514
	N1	1, 18.3	3.14	0.093	3, 47.2	53.96	<0.001	3, 47.2	2.19	0.101
	N2	1, 18.2	0.01	0.914	3, 47.2	71.87	<0.001	3, 47.2	0.86	0.470
	N3	1, 18.0	3.52	0.077	3, 46.8	23.53	<0.001	3, 46.8	3.12	0.035
	REM	1, 17.9	6.83	0.018	3, 46.7	37.36	<0.001	3, 46.1	0.85	0.473
	WASO	1, 18.6	4.58	0.046	3, 48.0	18.40	<0.001	3, 48.0	0.97	0.413
	Quality	1, 17.1	0.11	0.076	3, 46.3	0.34	0.798	3, 46.3	0.18	0.908

Group—SDB (*n* = 8), non-SDB (*n* = 12); Night—Baseline, Experimental Night 1, Experimental Night 2, Recovery; TIB—Time in Bed (min); ODI—Oxygen Desaturation Index; TST—Total Sleep time (min); SOL—sleep onset latency (min); ROL—REM onset latency (min); N1—Stage N1 minutes, N2—Stage N2 minutes; N3—Stage N3 minutes; REM—Rapid Eye Movement Sleep (min); WASO—Wake after sleep onset (min); Quality—Subjective sleep quality.

### 3.1. Study Variables

For ODI there was a significant main effect of *group* with ODI significantly higher across all sleeps in SDB group compared to non-SDB group. While TIB was set at 480 min for the Control and Recovery sleeps and 240 min for the two Experimental Sleeps, there were occasions where TIB did not match this exactly. Analyses revealed a significant main effect of *group* and a significant *group* × *night* interaction for actual TIB with the SDB groups obtaining an average of 9 min longer TIB than non-SDB group in the baseline sleep.

### 3.2. Sleep Variables

There was a main effect of *group* for TST (*p* = 0.05), REM, WASO and Arousals/h. The SDB group had less TST and REM and greater WASO compared to the non-SDB group. The *group* × *night* interaction was significant only for N3 with those in the SDB group obtaining 42 min less on the Baseline night.

The main effect of *night* was significant for all sleep variables except subjective sleep quality. Pairwise comparisons showed a reduced SOL and ROL and less TST, N1, N2, N3, REM and WASO on restricted nights compared to the Baseline and Recovery nights. Sleep Efficiency was lower during the Baseline sleep compared to all other sleeps.

**Figure 1:**
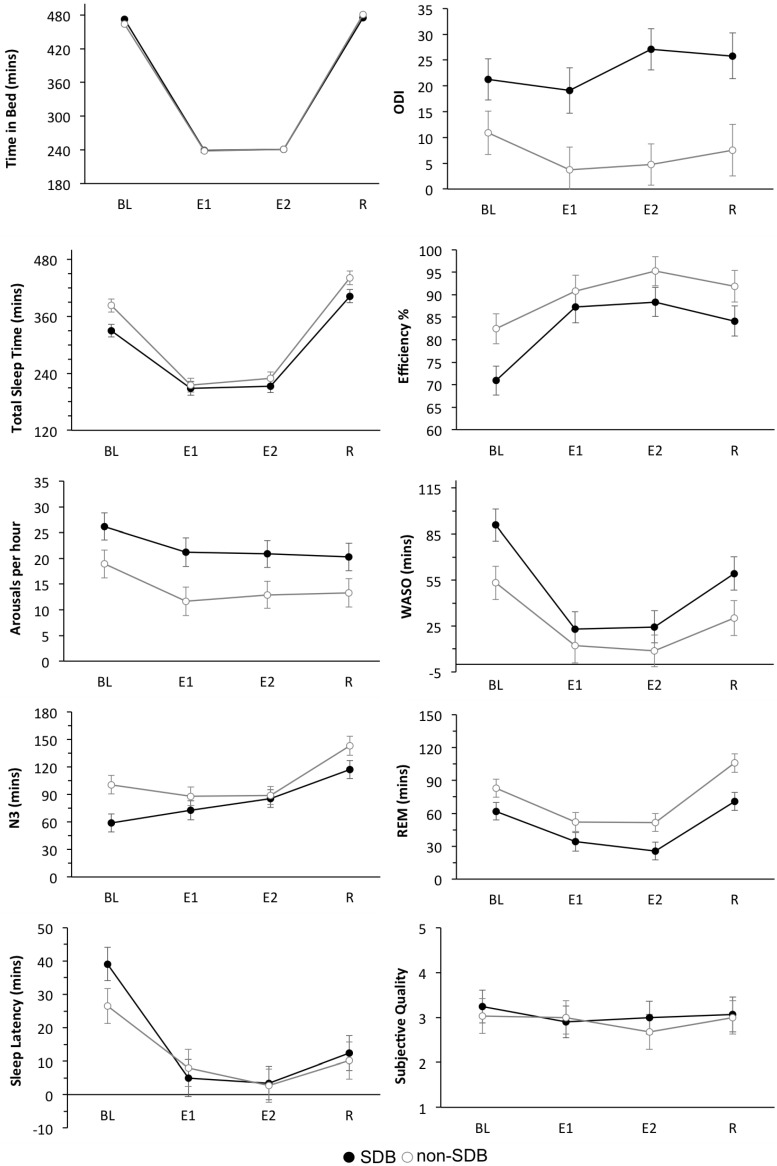
Sleep Variables. Mean ± Standard error or measurement (SEM) for TST, Efficiency, Arousals/h, WASO, N3, REM, SOL, Subjective Sleep Quality for the two groups: SDB, non-SDB and across the 4-nights: Baseline (BL), Experimental Night 1 (E1), Experimental Night 2 (E2) and Recovery (R).

Mean ± SEM values for the following sleep variables are shown in Figure 1. (TST, Efficiency, Arousals/h, WASO, N3, REM, SOL, Subjective Quality).

### 3.3. Wake Variables

For RRT, the main effects of *day* (F_2, 207_ = 38.6, *p* < 0.001) and *time* (F_3, 207_ = 3.9, *p* = 0.01) and the *day* × *group* interaction (F_2, 207_ = 5.2, *p* = 0.006) were significant. Pairwise comparisons showed that in the SDB group, RRT performance each day was significantly different to the other two days. In the non-SDB group, RRT performance on the experimental days was significantly worse compared to baseline but not to each other.

There was a significant main effect of *day* (F_2, 207_ = 11.3, *p* < 0.001) for Lapses with pairwise comparisons showing a significant (*p* < 0.05) increase in lapses across days.

**Figure 2:**
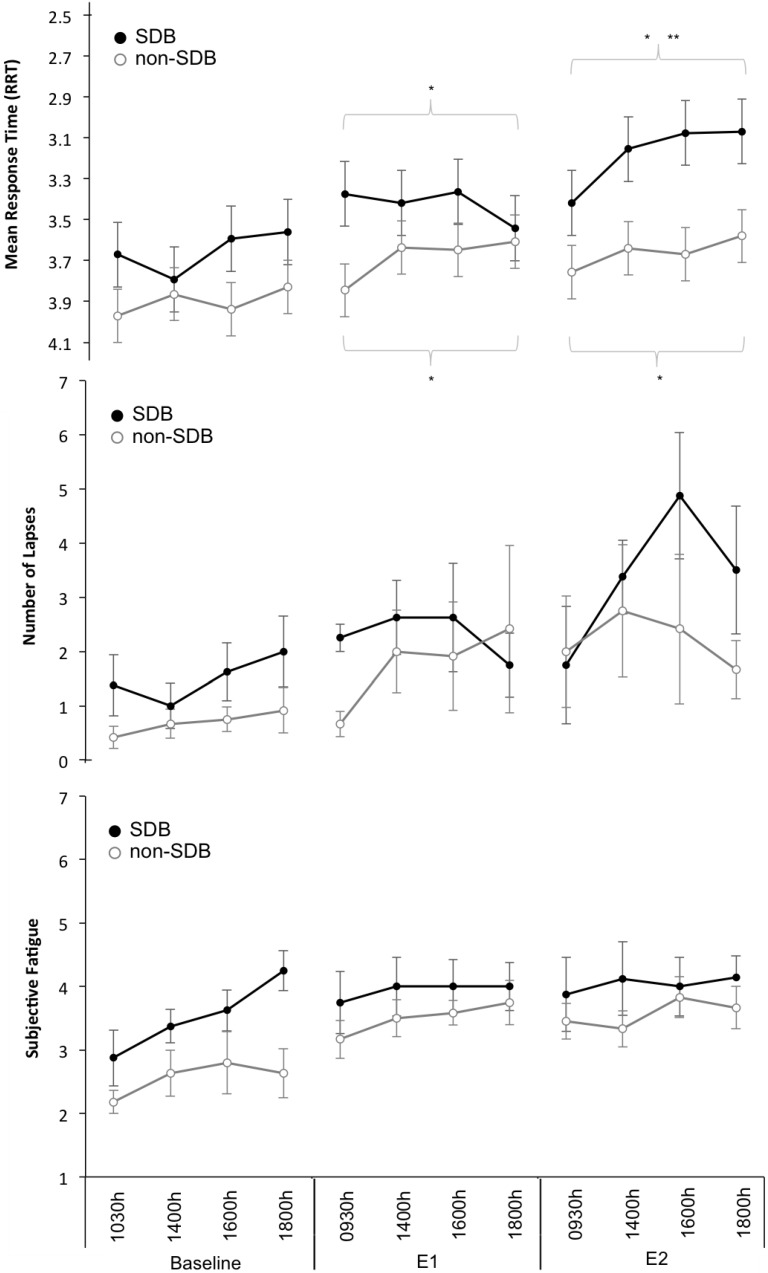
Wake Variables. Mean ± Standard error or measurement (SEM) for (top panel to bottom panel): Response time (reciprocal transformation used, RRT and scale inverted). Lapses (Response Times >500 ms) and Subjective Fatigue Ratings across the 3-days: Baseline, Experimental Day 1 (E1), Experimental Day 2 (E2) and across Time: 0930/1030, 1400, 1600, 1800. * Denotes significant difference compared to Baseline Day; ** Denotes significant difference compared to Experimental Day 1.

For Subjective Fatigue the main effects of *day* (F_2, 200.3_ = 21.1, *p* < 0.001) and *time* (F_2, 200.1_ = 5.2, *p* = 0.002) were significant. Pairwise comparisons showed fatigue on the Baseline Day was significantly (*p* < 0.05) lower compared to fatigue on the Experimental Days. Figure 2 illustrates mean ± SEM values for the wake variables across each day of the study.

## 4. Discussion

This study investigated differences in sleep and daytime function between SDB and non-SDB identified participants who participated in a 3-day-4-night fireground simulation that included two nights of sleep restriction (4 h). We hypothesised that objective measures of sleep would reflect changes commonly seen in individuals with untreated OSA. As predicted total sleep in the SDB group was less, and consisted of more wakefulness, more arousals, less SWS (N3) and less REM. Notably, participants in the SDB group were older and had a higher mean BMI than the non SDB group. While both these factors place them at an increased risk of OSA [8], these factors in and of themselves could have contributed to the differences between the two groups. Age related changes to sleep are well documented with reduced total sleep, efficiency, REM and SWS all part of normal aging [25]. In terms of BMI, overweight participants may have had greater difficulty getting comfortable on the stretcher beds, which were a standard size. Nonetheless, the differences in sleep [7] and importantly their ODI values suggest that an underlying SBD was highly probable [15,18].

We hypothesised that those in the SDB group would exhibit signs of increased vulnerability to the sleep restriction, namely increased self-reported fatigue, poorer response time performance and more lapses. While the SDB participants did not rate fatigue as higher or have more lapses, their response time performance was worse compared to those in the non-SDB group. Given the differences in sleep, this result is not surprising. In both groups, response time performance was worse on Experimental Days (following sleep restriction) compared to Baseline but where performance in the non-SDB group was maintained, performance in the SDB group continued to decline after the second restriction night. This finding is consistent with research showing that even when treated, individuals with SDB (specifically OSA) are more vulnerable to the performance effects of sleep restriction [12].

From a practical standpoint, the continued decline in RRT performance in the SDB group, is concerning. This simulation involved two nights of sleep restriction (4 h) but in the real-world, deployments can last for much longer [26]. Based on data from laboratory studies [1,2], performance would continue to decline across longer periods of sleep restriction (up to a week or more). Having significant numbers of volunteers with untreated SDB participating in a given multi-day deployment could have safety consequences. Importantly however, for these analyses, daytime performance on only one task was reported and while the PVT is considered a reliable assay of sleepiness/fatigue [22] it is not the only task relevant to fireground performance. Memory, and hand-eye coordination in addition to the huge physical demand all play important roles in effective firefighting. Driving performance may be of particular interest given that there is typically a drive, that can last up to 2–3 h, to and from the fireground [3]. The specific consequences of untreated SDB for all operational tasks needs to be assessed before the overall impact on fireground performance can be understood.

The mismatch between subjective and objective measures in the SDB group is also of interest and mirrors what has been found in large-scale sleep restriction studies in the laboratory [2]. While daytime sleepiness is a common symptom of SBD, particularly OSA, large variability between individuals has been reported [10,27,28]. Notably, pre-study ESS [13] scores (for both groups) were well below 10, (cutoff indicative of excessive daytime sleepiness) suggesting that subjective symptoms may not have been especially prevalent in this group [13]. Data collected during the study supports this because while subjective fatigue (for all participants) was greater following sleep restriction compared to baseline, mean values were still in the mid-range (4) of the scale (1–7) suggesting that fatigue levels overall weren’t extreme. We acknowledge that fatigue (as measured in this study) and sleepiness, are distinct constructs [29] and perhaps a sleepiness scale may have yielded different results. However, the two constructs are inherently interrelated and often used interchangeably [29].

Finally, while not measured in this investigation, impairment suffered by individuals with SDB might carry-over into their recovery. Visual inspection of the data shows a 52 min and 39 min difference in the “unrestricted” 8 h (Baseline and Recovery) sleeps between groups. From this, we would assume that sleep in the SDB group upon returning home would also be less. Given that their impairment (RRT) was significantly worse at the end of the campaign and that their sleep was of poorer quality, their ability to recover from the mental and physical exertion required during campaign bushfire suppression would be impacted. This has implications for their participation in domestic and occupational responsibilities and the appropriateness of them being redeployed. This is especially pertinent during the Fire Danger Season, where deployments may occur in quick succession.

### Limitations

This simulation was unique in that it combined elements of both laboratory and field research. Certain key variables were tightly controlled and/or manipulated (e.g., temperature, sleep times, daily protocol, meal times) but sleep in the days prior to the study were uncontrolled and participants were essentially unscreened. As such, there are possibly other factors that contributed to differences in sleep.

TIB was fixed for each night, but there were circumstances where, due to technical difficulties associated with the portable sleep laboratory, TIB values varied. As a result, those in the SBD group had longer TIB (9 min) on the Baseline night. This is not ideal but given that the overall difference on the baseline night was 52 min and that SBD group slept less despite the extra opportunity, we would not consider this to have influenced the overall results significantly.

Finally, the sample size was small (*n* = 20) and while oximetry is an appropriate proxy in the absence of nasal pressure and respiratory signals, it is not the “gold standard”.

## 5. Conclusions

Our retrospective investigation into the effects of suspected SDB on sleep and waking function during a fire ground simulation showed that in terms of sleep, SDB participants generally exhibited differences consistent with what is commonly seen in individuals with SDB. Importantly, these differences translated into next day performance with the SDB group exhibiting continued decline in response time performance with sleep restriction. While these results are of concern, further investigation into other relevant aspects of performance (mental and physical) is required in order to fully understand what impact untreated SDB has on volunteers in terms of their fireground work. At a minimum, we would recommend that education of SDBs and their risk factors and symptoms are made known across the relevant agencies to raise awareness among volunteers about their possible impact.
